# Diagnosis and management of drug-associated interstitial lung disease

**DOI:** 10.1038/sj.bjc.6602064

**Published:** 2004-08-31

**Authors:** N L Müller, D A White, H Jiang, A Gemma

**Affiliations:** 1 Department of Radiology, Vancouver General Hospital and University of British Columbia, Vancouver, British Columbia, Canada; 2 Memorial Sloan Kettering Cancer Center, New York, NY, USA; 3 AstraZeneca KK, Osaka, Japan; 4 Nippon Medical School, Tokyo, Japan

**Keywords:** interstitial lung disease, drug-associated ILD, lung cancer, NSCLC, algorithm

## Abstract

Symptoms of drug-associated interstitial lung disease (ILD) are nonspecific and can be difficult to distinguish from a number of illnesses that commonly occur in patients with non-small-cell lung cancer (NSCLC) on therapy. Identification of drug involvement and differentiation from other illnesses is problematic, although radiological manifestations and clinical tests enable many of the alternative causes of symptoms in advanced NSCLC to be excluded. In lung cancer patients, high-resolution computed tomography (HRCT) is more sensitive than a chest radiograph in evaluating the severity and progression of parenchymal lung disease. Indeed, the use of HRCT imaging has led to the recognition of many distinct patterns of lung involvement and, along with clinical signs and symptoms, helps to predict both outcome and response to treatment. This manuscript outlines the radiology of drug-associated ILD and its differential diagnosis in NSCLC. An algorithm that uses clinical tests to exclude alternative diagnoses is also described.

## INTRODUCTION

The diagnosis of drug-associated interstitial lung disease (ILD) involves three elements: clinical suspicion, differentiation from other parenchymal lung diseases using computed tomography (CT) and other clinical tests for alternative disease, and a compatible histological pattern. This review article will discuss the radiological evaluation of a patient with non-small-cell lung cancer (NSCLC) suspected of suffering from drug-associated ILD, the diagnosis and management of drug-associated ILD, and the development of a diagnostic algorithm designed to distinguish gefitinib (‘Iressa’)-associated ILD from other forms of parenchymal disease.

## SUSPICION OF DRUG-ASSOCIATED ILD IN PATIENTS WITH NSCLC

The onset of drug-associated ILD during therapy for advanced NSCLC usually occurs within a few weeks of the start of treatment (Thomas *et al*, 2000; Gupta *et al*, 2002; Kudrik *et al*, 2002; Read *et al*, 2002). Indeed, retrospective analysis of the first 152 patients in Japan to experience gefitinib-associated ILD showed that >75% of cases occurred within 3 months, with the majority of these occurring within 4 weeks.

The symptoms of drug-associated ILD, as with all forms of the condition, include rapidly developing breathlessness and a dry and unproductive cough, together with fever (Thomas *et al*, 2000; Gupta *et al*, 2002; Kudrik *et al*, 2002; Read *et al*, 2002). Such symptoms are nonspecific and can occur with a large number of common illnesses often associated with NSCLC, or they may be due to cancer progression or lung cancer therapy. These patients are also prone to pneumonia and many have radiation-related injury as a result of prior treatment. Cardiovascular causes of the symptoms, such as fluid overload, congestive heart failure and pulmonary embolus, are not uncommon. Differentiation of drug-associated ILD from these illnesses is difficult and the diagnosis is usually made by exclusion. High-resolution CT (HRCT) is recommended on first suspicion of ILD to provide an assessment of the parenchymal nature of the cause of symptoms.

## RADIOLOGY OF DRUG-ASSOCIATED ILD

The radiological manifestations of drug-associated ILD, although heterogeneous and nonspecific, enable many of the alternative causes of symptoms in advanced NSCLC to be excluded. There is no specific radiological pattern of parenchymal change connected with drug-associated ILD. Furthermore, in the early stages of disease, patients with symptoms secondary to drug reaction may have a normal chest radiograph.

High-resolution CT allows a more precise assessment of the presence, pattern and distribution of parenchymal and airway abnormalities than a chest radiograph. It has the advantage over lung biopsy of providing an overall view of the extent and pattern of parenchymal involvement rather than being limited to a small region, which may not be representative of the overall pattern of disease. However, there is limited information on the correlation between the findings on HRCT and histological patterns in drug-associated lung disease. Data based on a small number of cases suggest that the different histological patterns of drug reaction are not reflected by distinctive HRCT findings (Cleverley *et al*, 2002).

Despite these limitations, it seems reasonable to approach the radiological manifestations of drug-associated lung disease by the use of the underlying histological pattern (Ellis *et al*, 2000; Myers *et al*, 2003). Using such an approach, the most common manifestations can be classified into diffuse alveolar damage, hypersensitivity pneumonitis, organising pneumonia, nonspecific interstitial pneumonia (NSIP) and eosinophilic pneumonia (other less common drug reactions are not discussed here). Any one histological pattern can be caused by a number of different drugs. Furthermore, similar histology is found in other conditions that are not associated with drug use, such as idiopathic interstitial pneumonias, viral, bacterial and fungal pneumonia, pulmonary haemorrhage or leukaemia and collagen vascular disease. Occasionally, HRCT may demonstrate findings that are highly specific for the diagnosis, including increased attenuation in amiodarone lung and areas of fat attenuation in lipoid pneumonia.

### Diffuse alveolar damage

**Figure 1:**
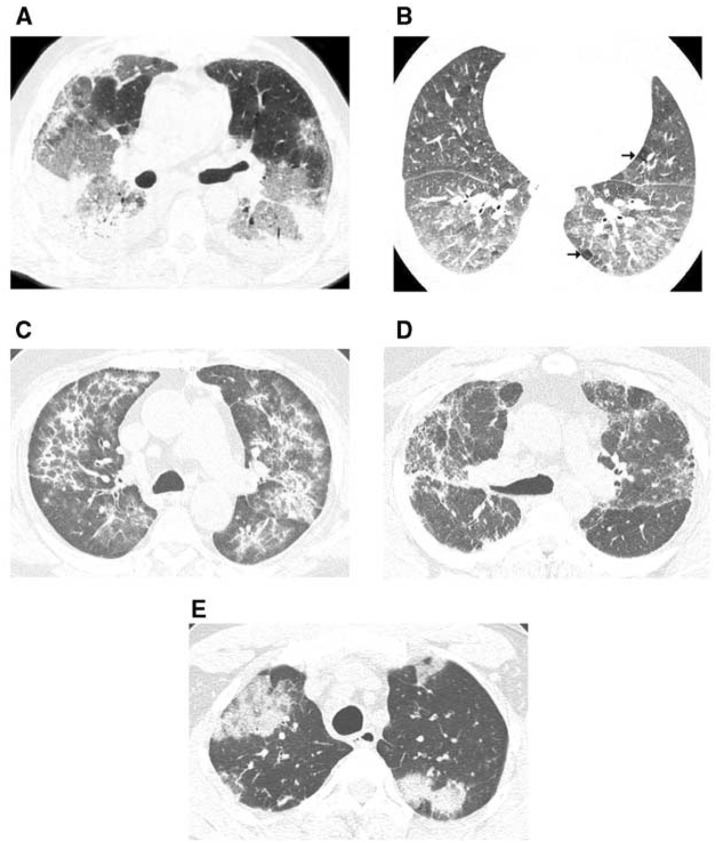
High-resolution CT images demonstrating radiology of drug-associated ILD. (**A**) A 77-year-old man with diffuse alveolar damage secondary to amidarone; note the extensive bilateral ground-glass opacities, airspace consolidation and bilateral pleural effusions. (**B**) A 36-year-old woman with hypersensitivity pneumonitis secondary to sertraline; note the extensive bilateral ground-glass opacities and lobular areas of air trapping (arrows). (**C**) A 69-year-old man with BOOP-like reaction to amiodarone; note the mild reticulation and bilateral areas of consolidation and ground-glass opacities in a predominantly peribronchial distribution. (**D**) A 47-year-old man with NSIP reaction to bleomycin; note the extensive bilateral ground-glass opacities with mild superimposed reticulation. (**E**) A 47-year-old man with eosinophilic pneumonia reaction to dilantin; note the patchy bilateral areas of consolidation involving the peripheral regions of the upper lobes.

Diffuse alveolar damage is characterised histologically by the presence of alveolar airspace and interstitial oedema, hyaline membrane formation and proliferation of type II pneumocytes (Rossi *et al*, 2000; Cleverley *et al*, 2002). In relation to drug-associated pulmonary disease, it occurs most commonly with cytotoxic agents such as bleomycin and, less commonly, with aspirin and narcotics (Rossi *et al*, 2000; Cleverley *et al*, 2002). The corresponding radiological features are also found in adult respiratory distress syndrome (ARDS). The chest radiograph shows bilateral patchy or homogeneous airspace consolidation involving mainly the middle and lower lung zones (Rossi *et al*, 2000; Cleverley *et al*, 2002). High-resolution CT demonstrates extensive bilateral ground-glass opacities and dependent areas of airspace consolidation (Rossi *et al*, 2000; Erasmus *et al*, 2002) (Figure 1A

).

### Hypersensitivity pneumonitis

A number of drugs may result in hypersensitivity pneumonitis, including methotrexate, cyclophosphamide and antidepressants such as fluoxetine and amitriptyline (Ellis *et al*, 2000). The radiological and HRCT findings are identical to those seen in hypersensitivity pneumonitis secondary to the inhalation of organic dust and consist of bilateral ground-glass opacities and/or small poorly defined centrilobular nodular opacities (Ellis *et al*, 2000; Cleverley *et al*, 2002). The majority of patients also demonstrate lobular areas of air trapping, although this is less apparent than in extrinsic allergic alveolitis to airborne agents (Ellis *et al*, 2000) (Figure 1B).

### Organising pneumonia

Organising pneumonia, also known as bronchiolitis obliterans organising pneumonia (BOOP)-like reaction, has been reported most frequently in association with methotrexate, cyclophosphamide, gold, nitrofurantoin, amiodarone, bleomycin and busulphan (Cleverley *et al*, 2002; Erasmus *et al*, 2002). The chest radiograph shows patchy bilateral areas of consolidation, masses or nodules, which may be symmetric or asymmetric (Müller *et al*, 1990; Ellis *et al*, 2000). In a few patients, the disease manifests with a lone mass. On HRCT, areas of consolidation often have a predominantly peripheral or peribronchial distribution (Müller *et al*, 1990; Ellis *et al*, 2000) (Figure 1C).

### Nonspecific interstitial pneumonia

Nonspecific interstitial pneumonia is one of the most common forms of drug-associated pneumonitis. Nonspecific interstitial pneumonia is characterised histologically by homogeneous alveolar wall thickening by fibrous tissue and mononuclear inflammatory cells. The reaction is seen in association with a variety of drugs, the most common being methotrexate, amiodarone and carmustine (Erasmus *et al*, 2002). The corresponding radiographical and HRCT findings usually consist of patchy or diffuse ground-glass opacities (Rossi *et al*, 2000; Erasmus *et al*, 2002) (Figure 1D). On disease progression, there may be evidence of fibrosis with development of a reticular pattern and traction bronchiectasis. In some patients, the fibrosis is patchy in distribution and predominantly peribronchovascular, a pattern most commonly seen in patients receiving nitrofurantoin. Late chemotherapy lung may predominate in the upper and lateral parts of the lung.

### Eosinophilic pneumonia

Eosinophilic pneumonia is characterised histologically by the accumulation of eosinophils in the alveolar airspaces and infiltration of the adjacent interstitial space by eosinophils and variable numbers of lymphocytes and plasma cells. Peripheral blood eosinophilia is present in ⩽40% of patients. Eosinophilic pneumonia secondary to drug reaction is seen most commonly in association with methotrexate, sulphasalazine, para-aminosalicylic acid, nitrofurantoin and nonsteroidal anti-inflammatory drugs. Chest radiography and HRCT show bilateral airspace consolidation, which tends to involve mainly the peripheral lung regions and the upper lobes (Rossi *et al*, 2000; Cleverley *et al*, 2002) (Figure 1E).

## DIFFERENTIAL DIAGNOSIS OF DRUG-ASSOCIATED ILD IN NSCLC

Alternative diagnoses to drug-associated ILD in NSCLC include progression of the cancer, infection, radiation-related lung injury, fluid overload, congestive heart failure and pulmonary embolus. Additionally, some lung cancer patients may develop BOOP or other steroid-responsive inflammatory disorders that cannot be clearly related to drug therapy. Other possible causes of dyspnoea that do not give infiltrates include comorbid diseases such as chronic obstructive pulmonary disease (COPD) and aspiration of food and saliva (particularly in patients with vocal cord paralysis or brain metastases).

In the USA, pulmonary embolism is particularly common in patients with lung cancer, with as many as 20% of patients estimated to develop a deep vein thrombosis or pulmonary embolism during the course of their disease (Lieberman *et al*, 1961; Sack *et al*, 1977; Lee and Levine, 2003).

Most episodes of pneumonia in patients with lung cancer are due to bacteria. This is particularly the case when risk factors of neutropenia, endobronchial lesions, underlying COPD and aspiration are present. Although opportunistic infections are not common, fungal infections should be considered if the patient has received a high dose of corticosteroids. Viral infections with herpes simplex, cytomegalovirus or respiratory syncytial virus may also rarely result in pneumonia in patients who have received high-dose corticosteroids or very intensive chemotherapy.

The development of lung fibrosis following radiation therapy is well documented and is usually confined to the radiation port (Abid *et al*, 2001; Aviram *et al*, 2001). With the use of three-dimensional radiation portals, however, resulting infiltrates from radiation may not result in the traditional straight-edged infiltrate and may be more difficult to distinguish from other entities.

### Investigations of dyspnoea in lung cancer patients

Patients with lung cancer who present with respiratory failure should undergo systematic investigation. Pulmonary function tests, such as measurement of forced expiratory volume in 1 s, carbon monoxide diffusing capacity and measurement of arterial oxygen saturation with pulse oximetry, are commonly used. These tests ascertain the type of defect, for example, obstructive or restrictive ventilatory defects, which helps establish the cause, such as an exacerbation of any underlying obstructive airways disease versus interstitial disease. They also indicate the severity of the disease that helps determine the need for further assessment and treatment.

A standard chest CT scan is commonly performed to exclude a diagnosis of disease progression or pulmonary embolism. However, as discussed previously, obtaining high-resolution cuts is very helpful (HRCT) if drug-associated ILD is suspected.

Bronchoscopy is useful to evaluate some NSCLC patients with dyspnoea to assess extension of the cancer and to exclude an opportunistic infection using bronchioalveolar lavage. However, the value of a bronchoscopy in establishing the diagnosis of drug-associated ILD is less clear, since bronchioalveolar lavage is not specific for drug-associated disease, and biopsies obtained by transbronchial biopsy are small and often do not yield enough tissue to make this distinction. Open lung biopsy is rarely performed in NSCLC patients with respiratory distress since most patients have advanced disease with a limited prognosis. Furthermore, procedures requiring surgery are not usually believed to arrive at a definitive diagnosis of drug-associated ILD.

Patients with mild symptoms or pulmonary function abnormalities (such as a decrease in diffusing capacity of <20% from baseline or no change in oxygen desaturation during exercise), or with transitory or slight radiographical infiltrates are monitored using pulmonary function tests, symptoms and usually CT scans. Diagnostic evaluation and treatment for drug-related lung disease is considered in those patients who experience dyspnoea at rest or on mild exertion, have a ⩾20% decrease in carbon monoxide diffusing capacity, or experience oxygen desaturation at rest or during exercise. Patients whose radiographical infiltrates are extensive or progressive are also considered for therapy.

## DIAGNOSTIC ALGORITHMS FOR ILD IN PATIENTS WITH NSCLC

Retrospective analysis of the adverse-event reports from patients diagnosed with ILD following gefitinib treatment is also difficult as there is often limited or heterogeneous clinical information, no pathology result and no access to the results of radiological investigations.

A diagnostic algorithm has therefore been developed to assess the accuracy of the reports of drug-associated ILD among Japanese patients receiving gefitinib. This approach to differential diagnosis used an algorithm developed to aid early diagnosis of drug-associated ILD in clinical practice.

**Figure 2:**
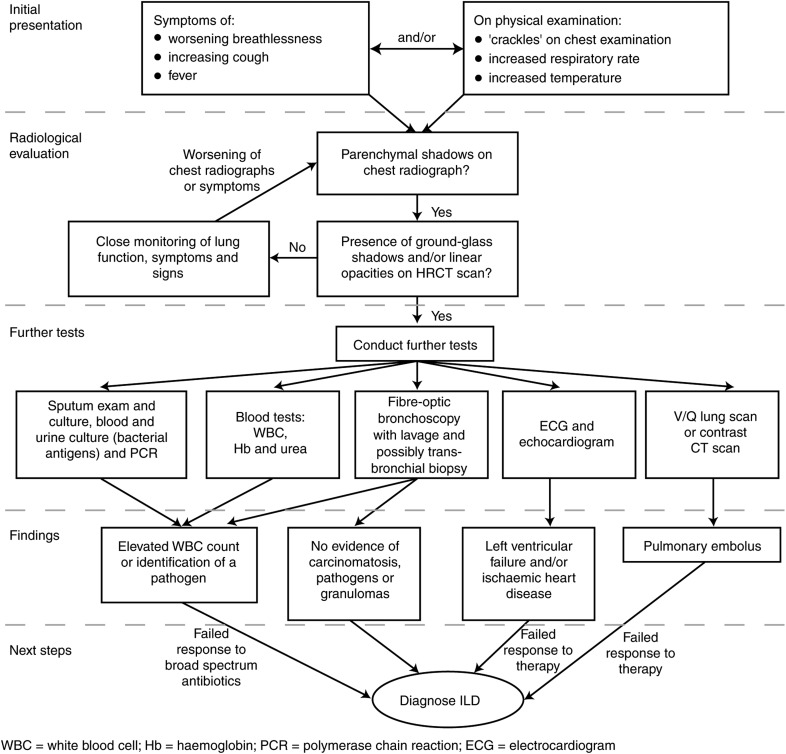
Diagnostic algorithm of gefitinib-associated ILD in Japanese patients with NSCLC.

The algorithm used both radiological and clinical evidence to exclude alternative diagnoses, such as infection, tumour progression, heart failure and pulmonary embolism, to arrive at a presumptive diagnosis of drug-associated ILD (Figure 2

; Table 1

**Table 1:** Nonradiological tests for ILD

**Alternative diagnosis**	**Test**
Infection	Erythrocyte sedimentation rate
	C-reactive protein levels
	White blood cell count and differential count of leucocytes
	Sputum/blood cultures
	Serum antibody tests for pathogens, for example, fungal infection, beta-D-glutan, clamydia, cytomegalovirus, etc
	PCR in blood, other body fluids, etc
	
Cancer	Tumour markers
progression	Changes in pleural effusion and pericardial effusion
	Biopsy evidence
	
Association	KL-6, SP-A, SP-D or serum soluble interleukin-2 receptor levels
with ILD	LDH levels
	Respiratory function tests: SpO_2_, with or without exercise; %vital capacity, FEV_1_/forced vital capacity; and %DLCO
	Bronchioalveolar lavage combined with fibrescopic transbronchial lung biopsy
	
Renal failure	Blood urea nitrogen levels
	Creatinine levels
	Sodium, potassium and chloride levels
	
Heart failure	Electrocardiogram for myocardial infarction
	Reduced left ventricle ejection fraction on echocardiogram
	
Anaemia	Red blood cell count
	Haemoglobin levels
	
Pulmonary	Proximal obstruction of pulmonary artery on contrast CT scan
embolus	Perfusion lung scan, lung scintigraphy
	Blood tests for thrombophilia: platelet count, fibrinogen degradation product levels, prothrombin time and activated partial thromboplastin time
	LDH levels
Histology from lung biopsy or specimens obtained at autopsy	

PCR=polymerase chain reaction; LDH=lactate dehydrogenase; SpO_2_=arterial oxygen saturation with pulse oximetry; FEV_1_=forced expiratory volume in 1 s; DLCO=carbon monoxide diffusing capacity and CT=computed tomography.

). Patients were categorised based on the strength of evidence supporting the diagnosis of drug-associated ILD (category 1, good supporting evidence for ILD; category 2, limited supporting evidence for ILD; category 3, no supporting evidence for ILD).

### Retrospective use of the algorithm

In the first assessment, adverse-event reports from Japanese patients receiving gefitinib for NSCLC were evaluated using the algorithm, with available clinical information and radiographical reports but without access to chest radiographs or HRCT films. These findings were then compared with those of a second assessment, conducted by an independent panel of expert radiologists and physicians. The panel assessed the radiological and clinical findings for the same patient population using a set of standardised criteria (Cleverley *et al*, 2002).

The initial 152 reported patients with NSCLC in Japan who had experienced adverse events involving the lungs while receiving treatment with gefitinib were included in the first assessment of the algorithm. Of these, 135 were included in the second assessment because radiological examinations, including 47 with CT imaging, were available. Approximately 20% of these patients (23 out of 135) were considered by the expert panel not to have drug-associated ILD, highlighting the difficulty in diagnosing drug-associated ILD in patients with NSCLC.

These results were then compared with those obtained using the algorithm. A large proportion (17 out of 23) of patients not considered by the panel to have drug-associated ILD had been categorised as having ‘good’ or ‘limited’ supporting evidence for drug-associated ILD. The initial algorithm based on the terminology used in radiology reports, without considering differential diagnosis criteria, was not adequate. Infection, heart failure and tumour progression can be differentially diagnosed and excluded using additional radiological clinical data.

Therefore, the algorithm is now used to enable the clinician to make a diagnosis having first undertaken all the necessary steps in the clinical examination and investigation. This approach is applied to the prospective nested case–control study to investigate the relative risk and risk factors for ILD in NSCLC patients in Japan treated with and without gefitinib. An independent case review board will review each reported case of ILD using the information gathered from the algorithm.

## MANAGEMENT OF ACUTE RESPIRATORY DISTRESS IN PATIENTS WITH LUNG CANCER

General principles in the management of acute respiratory distress in patients with lung cancer are influenced by the multiple causes of respiratory failure that are associated with cancer, lung cancer therapy and the presence of comorbid disease. In addition, the diagnosis may remain uncertain, even when invasive procedures are performed, and empirical therapy for the likely causes is frequently given. Finally, respiratory failure in patients with cancer results in high mortality (Groeger *et al*, 1999) requiring aggressive assessment and therapy.

### Treatment of drug-associated ILD

There are no firm guidelines for the treatment of drug-associated ILD and therapy tends to be on an empirical basis. Withdrawal of the drug suspected of causing the ILD is the first step in treatment. For patients in respiratory failure, high-dose methylprednisolone (250 mg four times a day i.v.) for several days is commonly used. If the patient responds, then the dose is reduced (0.5–1 mg kg^−1^ day^−1^ orally) for several weeks before being gradually tapered. For patients in respiratory distress, methylprednisolone (1 mg kg^−1^ day^−1^ or 60 mg day^−1^) is commonly used, again with gradual dose reduction. Low-dose methylprednisolone (10–20 mg) is prescribed for patients with mild radiographical or pulmonary function abnormalities, particularly if oral corticosteroids are contraindicated (White and Stover, 1984; Baughman *et al*, 1994; American Thoracic Society, 2002).

Immunosuppressive agents such as azathioprine have been used as steroid-sparing agents in the treatment of drug-associated ILD, particularly in chronic cases of bleomycin-associated ILD (Maher and Daly, 1993). These agents are useful for patients in whom corticosteroids cannot be tapered or who cannot tolerate corticosteroids. It is also advisable to avoid combining agents associated with ILD, such as bleomycin and possibly mitomycin, with other agents that cause lung damage, such as oxygen and radiation, as this may result in worsening of the lung damage. If radiotherapy is indicated in a patient who has experienced mild bleomycin toxicity, then concomitant low-dose corticosteroid may minimise any further lung damage. Following resolution of the drug-associated ILD, some patients are susceptible to exacerbations on subsequent insults (e.g. during a respiratory infection) and may require further treatment.

In cancer patients, drug-associated ILD is most commonly observed during mitomycin, paclitaxel, docetaxel or gemcitabine therapy. Table 2

**Table 2:** Common types of lung damage during chemotherapy and their response to treatment

**Agent/lung damage**	**Therapy**	**Response**
*Mitomycin*		
Acute pneumonitis (interstitial and noncardiac oedema)	Methylprednisolone 250 mg every 6 h for 2–3 days followed by prednisone 0.5 mg kg^−1^ for 6 weeks with gradual dose reduction	Partial
Chronic pneumonitis	Prednisone 60 mg four times a day for 6 weeks followed by gradual dose reduction	Fair
		
*Paclitaxe*l		
Infusion hypersensitivity	Pretreatment with an antihistamine, a corticosteroid and a histamine	Preventative
Interstitial pneumonitis	H2-blocker Observation or corticosteroids	Good^a^
		
*Docetaxel*		
Fluid retention syndrome	Pretreatment with dexamethasone	Good^b^
Interstitial pneumonitis	Corticosteroids	Fair^a^
ARDS-like pattern	Corticosteroids	Fair^a^
		
*Gemcitabine*		
Dyspnoea	None	Good
ARDS (mild capilliary leak)	Corticosteroids, with continuation of gemcitabine therapy in some cases	Good
Interstitial pneumonitis in combination with docetaxel or paclitaxel)	Corticosteroids	Variable

Buzdar *et al* (1980), Chang *et al* (1986), Goldberg and Vannice (1995), Rivera *et al* (1995), Ramanathan and Belani (1996), Bookman *et al* (1997), Merad *et al* (1997), Piccart *et al* (1997), Semb *et al* (1998), Vander Els and Miller (1998), Dunsford *et al* (1999), Thomas *et al* (2000), Fogarty *et al* (2001) and Read *et al* (2002).

a Even when paclitaxel is used in conjunction with radiotherapy.

b When docetaxel is used in combination with radiotherapy or gemcitabine, the response is variable to poor.

outlines the types of injury reported with these agents and their response to therapy (Buzdar *et al*, 1980; Chang *et al*, 1986; Goldberg and Vannice, 1995; Rivera *et al*, 1995; Ramanathan and Belani, 1996; Bookman *et al*, 1997; Merad *et al*, 1997; Piccart *et al*, 1997; Semb *et al*, 1998; Vander Els and Miller, 1998; Dunsford *et al*, 1999; Thomas *et al*, 2000; Fogarty *et al*, 2001; Read *et al*, 2002; Barlesi *et al*, 2004). Acute pneumonitis, both interstitial and noncardiac pulmonary oedema pattern, has been observed with mitomycin. Response to high-dose methylprednisolone has been reported within 24–48 h of therapy; however, approximately 60% of patients developed ongoing and persistent lung disease (Rivera *et al*, 1995). Chronic pneumonitis, similar to bleomycin-type pneumonitis, has also been reported during mitomycin therapy, which responded to prednisone therapy (Buzdar *et al*, 1980; Chang *et al*, 1986). As with bleomycin, it has been suggested that oxygen therapy be avoided during mitomycin therapy, although the evidence for this is not as established as that for bleomycin (Klein and Wilds, 1983).

Infusion hypersensitivity is very common during paclitaxel therapy. Pretreatment with an antihistamine, a corticosteroid and an H2 blocker largely prevents or ameliorates this reaction (Bookman *et al*, 1997). Mild interstitial pneumonitis with transitory infiltrates has also been reported; observation or low-dose corticosteroids resulted in a good response, even when this occurred in conjunction with radiotherapy (Goldberg and Vannice, 1995; Ramanathan and Belani, 1996).

Fluid retention syndrome during docetaxel therapy is very common and is associated with dyspnoea. This reaction can be prevented by dexamethasone (Piccart *et al*, 1997; Semb *et al*, 1998). Interstitial pneumonitis and ARDS-like pattern have also been reported with docetaxel, particularly when it is used in combination with radiotherapy or gemcitabine. Both reactions respond well to corticosteroid therapy; however, when docetaxel is used in combination with radiotherapy or gemcitabine, the response to corticosteroid therapy can be variable to poor, with some deaths reported (Merad *et al*, 1997; Dunsford *et al*, 1999; Read *et al*, 2002).

Self-limiting dyspnoea is common during gemcitabine therapy. Additionally, many patients experience a mild capillary leak throughout the lung, similar to that seen in ARDS. However, these patients are asymptomatic and have normal pulmonary function; therefore, gemcitabine therapy can be continued without the need for corticosteroids. Gemcitabine-associated ILD has also been reported and the response to corticosteroid therapy is variable. As with docetaxel, more deaths due to ILD have been reported when gemcitabine is used in combination with other therapies (radiotherapy or chemotherapy) (Vander Els and Miller, 1998; Dunsford *et al*, 1999; Thomas *et al*, 2000; Fogarty *et al*, 2001; Barlesi *et al*, 2004).

### Re-challenge of drug therapy in suspected cases of ILD

In the management of patients with NSCLC, it is often necessary to treat patients with an agent that has been previously associated with ILD. In this scenario, it is important to consider the patient's previous response to that therapy, the severity of the lung damage and the respiratory distress, the presence of fibrosis and the previous response to corticosteroid therapy. For some patients, the benefits may outweigh the risks and therapy may be re-instituted with concomitant low-dose prednisone (10–20 mg day^−1^); however, for some patients and/or agents the potential for causing further lung damage may be too great.

## SUMMARY

In summary, lung cancer patients receiving systemic therapy frequently develop dyspnoea and infiltrates. It is difficult to make a specific diagnosis in most cases because of the difficulty of performing invasive procedures in this patient population. Radiological assessment, and HRCT in particular, can play a key role in establishing a diagnosis of drug-associated ILD; however, in the vast majority of cases, the radiological manifestations of drug-associated pulmonary injury are nonspecific, making an accurate diagnosis difficult. Corticosteroids are indicated for suspected drug-associated ILD; however, the outcome is variable unless the patient develops respiratory failure, in which case the mortality is high.

As a result, clinicians are reluctant to withhold corticosteroid therapy if there is any indication of drug toxicity, further complicating the diagnosis. However, once a patient responds to corticosteroid therapy, the decision to re-institute a drug suspected of causing ILD is made on an individual basis. In this article we described an algorithm developed to assess the incidence of drug-associated ILD in Japanese patients receiving gefitinib for NSCLC. Such an algorithm, once validated, may be a useful tool in the differential diagnosis of ILD in patients with cancer and help clarify some of the apparent discrepancies in the incidence and reporting of ILD.
